# EVALUATION OF THE RESCUE PROGRAM FOR REDUCING STROKE CAREGIVER STRESS AND IMPROVING PROBLEM-SOLVING ABILITIES

**DOI:** 10.1093/geroni/igz038.409

**Published:** 2019-11-08

**Authors:** Meggan Jordan, I Magaly Freytes, Tatiana Orozco, Stuti Dang, Constance R Uphold

**Affiliations:** 1 California State University Stanislaus, Turlock, California, United States; 2 North Florida/South Georgia Veterans Health System, Gainesville, Florida, United States; 3 Miami Veterans Affairs Healthcare System- GRECC, Miami, Florida, United States

## Abstract

Research shows that caregiver interventions that combine problem-solving with psycho-education are the most effective for addressing stroke caregiver concerns. The Resources and Education for Stroke Caregivers’ Understanding and Empowerment (RESCUE) program, designed to reduce caregiver stress, depression, and burden, developed as a result of this evidence. A 4-week telephone and web-based clinical demonstration project led by registered nurses was established as part of the VHA Office of Geriatrics and Extended Care’s Non-Institutional Long Term Care Initiative. The goals of this clinical demonstration were to improve problem-solving skills and provide individualized support for stroke caregivers. A single-group pre and post-test design was used and 72 caregivers of veterans with stroke completed the intervention; qualitative and quantitative methods were used for evaluation. The outcome variables were caregiver depressive symptoms, problem-solving abilities, burden, health-related quality of life and care recipient functional abilities. Post-tests were conducted 2-6 weeks after the intervention. The evaluation found that there were statistically-significant decreases in caregiver depressive symptoms and burden from pre- to post-test assessments. Caregivers’ negative problem orientation significantly decreased. The other components of problem-solving abilities did not change. Qualitative data revealed how the program increased caregivers’ confidence in problem-solving which led to new strategies to relieve stress. Interviews also revealed how the intervention affected caregivers in unexpected ways, such as improved intimate relationships and new perspectives on caregiving. The preliminary effectiveness and barriers and facilitators of implementing a stroke caregiver program will be discussed.

